# Oligomers of diphenylalanine examined using cold ion spectroscopy and neural network-based conformational search†

**DOI:** 10.1039/d4cp03476g

**Published:** 2024-10-16

**Authors:** Vladimir Kopysov, Ruslan Yamaletdinov, Oleg V. Boyarkin

**Affiliations:** a SCI-SB-RB Group, ISIC, École Polytechnique Fédérale de Lausanne 1015 Lausanne Switzerland oleg.boiarkin@epfl.ch

## Abstract

Diphenylalanine (Phe_2_) is the primary building block of many self-assembling nanostructures that are important in biology and materials science. Understanding the detailed mechanism of their formation requires knowledge of the structural motives that the smallest oligomers attain at the very first steps of the process. Herein, we first employed high-resolution mass spectrometry to assign protonated Phe_2_ and its 2–13-unit oligomers formed in the gas phase from solution *via* electrospray ionization and then used cold ion spectroscopy to record UV and IR spectra for the monomer, dimer and hexamer. UV spectroscopy suggests the likely lack of specific strong proton–π interactions in oligomers larger than octamers, implying their certain structural stabilization. IR spectroscopy and quantum chemical calculations, enhanced by neural network-based conformational search, jointly determined the lowest-energy structures of the Phe_2_ monomer and dimer.

## Abstract 2

Cold ion spectroscopy coupled with mass spectrometry and quantum chemical calculations enhanced by neural network search reveal 3D structures of Phe_2_ oligomers.

## Graphical abstract

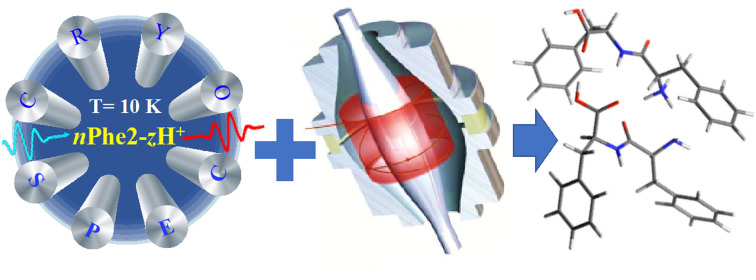

## Introduction

Peptide and protein aggregation into oligomers and fibrils in living organisms is often associated with amyloidosis and other disorders such as Alzheimer's and Parkinson's diseases.^1,2^ Phenylalanine-based peptides own a particularly high propensity for self-assembly of ordered supramolecular structures. For instance, phenylalanine (Phe) amino acid was shown to form toxic fibrils in phenylketonuria,^3^ while diphenylalanine (Phe_2_) is considered the key structural motif of Alzheimer's β-amyloid aggregates.^4,5^ Moreover, the capability of Phe-based peptides to self-assemble into nanostructures with various morphologies (*e.g.*, spheres, sheets, fibers, and wires) and remarkable physical properties is exploited in materials science.^6^ Hollow nanotubes formed from Phe_2_ dipeptides, for instance, demonstrate metal-like stiffness with a Young's modulus of 27 GPa,^7^ while Boc-Phe_2_ (Phe_2_ with the N-terminus protected by a tert-butoxycarbonyl group) self-assembles into even more rigid nanospheres.^8^ It was also shown that nanoscale materials produced from Phe_2_ or similar peptides exhibit unique optical properties of zero-dimensional quantum dots or two-dimensional quantum wells and good piezoelectric properties.^9^

At the micro- and nano-scale, structures of Phe_2_ assemblies have been extensively studied experimentally. X-Ray crystallography revealed that the Phe_2_ monomer crystallizes in the form of hexagonal units that stack upon each other to form a helical structure with the inner channel filled with water.^10^ In the hexagon, the dipeptides are arranged in the head-to-tail manner, thus establishing a network of strong hydrogen bonds between the charged N- and C-termini as well as between these groups and the first layer of water molecules confined in the channel. The bulky side chains of Phe_2_, on the contrary, point away from the channel and constitute the outer surfaces of individual helical structures, which can be “glued” together through hydrophobic and proton–π interactions into larger supramolecular assemblies. The morphology of the resulting nanostructures, studied using scanning and transmission electron microscopy, optical microscopy, and other techniques, appeared to significantly depend on the conditions used to produce them.^5,6^ Rational choice of these conditions, and understanding the reasons for amyloid formation in living organisms, require knowledge of the very first steps of Phe_2_ oligomer formation, which, thus far, remains obscure.

Different molecular dynamics (MD) simulations^11–14^ concur that the self-assembly of Phe_2_ involves formation of small oligomers at the early stage but diverge on the representative structures of the intermediates. Validation of the proposed theoretical models by conventional condensed-phase techniques remains challenging. For instance, nuclear magnetic resonance (NMR) analysis of Phe_2_ nanotube self-assemblies showed that this is likely a nucleation-dependent process.^15^ A later study by time-resolved Raman microscopy suggested a two-step model for the kinetics of the process, with the assemblies up to hexamers being formed at the first, nucleation stage;^16^ however, the structure of the intermediates still has not been elucidated due to their structural diversity.

Small oligomers of Phe and Phe_2_ were also studied in the gas phase, where the elimination of solvent drastically reduces their structural heterogeneity. For instance, Do *et al.* employed a combination of mass spectrometry (MS) and ion mobility spectrometry (IMS) to structurally characterize oligomers of Phe.^17^ The results suggest that the small aggregates of Phe are built of tetrameric core blocks, which stack on top of each other to form a tube. The aggregates larger than 20-mers are, likely, formed by two or even four tubes that grow parallel to each other and are bound by T-shaped π-stacking.

Infrared (IR) laser spectroscopy is another powerful technique that is widely used for the structural determination of gas-phase molecules and ions. It provides characteristic vibrational fingerprints, which are fundamentally determined by 3D molecular structure. In particular, similar IR multiple photon dissociation (IRMPD) spectra of the protonated room-temperature Phe_2_ monomer were independently recorded by two groups.^18,19^ Lepere *et al.* also performed quantum chemical calculations and compared the predicted vibrational transitions of several low-energy conformers of [Phe_2_ + H]^+^ against the experimentally measured spectrum.^19^ The comparison showed that the vibrational transitions of, at least, two conformers reasonably match the IR spectrum; however, an insufficient spectral resolution did not allow choosing between them. Several groups have recorded conformer-selective IR spectra of the neutral Phe_2_ monomer and identified the 3D structure of its two conformers by comparing the experimental spectra with theoretically calculated ones.^20–22^ Both ultraviolet (UV) and IR spectra of the Phe_2_ dimer appeared, however, too broad (this was attributed to insufficient cooling of the dimer in the molecular beam) to allow a reliable determination of its 3D structure.^22^

Ionized biomolecules nowadays can be routinely cooled to vibrational temperature as low as 10 K using cryogenic ion traps.^23,24^ The cooling drastically improves vibrational resolution in UV and IR spectra,^25^ which makes spectroscopic fingerprints of the ions extremely sensitive even to tiny differences in their 3D structures. In addition, the use of IR-UV double-resonance spectroscopy allows for recording vibrational spectra of single conformers.^26,27^ This further improves the spectral resolution, making validation of the calculated structures more reliable. Over the years, a combination of cold ion spectroscopy (CIS) with high-level quantum chemical calculations has been successfully employed for stringent validation of the 3D structure of small to midsize biomolecules,^25^ as well as for structural characterization of large non-covalent complexes, including, for instance, oligomers of an 11-residue amyloidogenic peptide.^28^

Our study herein aims to provide a structural assessment of the smallest protonated non-covalent complexes of Phe_2_ in the gas phase. Despite all the developments, solving intrinsic structures of the midsize to large non-covalent complexes, such as oligomers of Phe_2_, remains challenging for both spectroscopy and theory. The difficulty originates from the large number of atoms, non-covalent bonds, often, high structural flexibility of these species and the existence of several possible protonation sites. The resulting high conformational heterogeneity makes achieving a vibrational resolution difficult for the experiments. It also makes challenging for the theory to perform the conformational search of the lowest energy structures among myriads of computer-generated candidate molecular geometries. In this respect, one of the objectives of this work was to evaluate how large non-covalent complexes still can be treated by the experiment and whether their structures can be solved at a readily affordable level of computational power by the quantum chemical calculations that include a recently tested for the molecules neural network (NN) approach.^29,30^

Herein, protonated Phe_2_ and its non-covalent complexes with up to 13 units are interrogated using IR/UV cold ion spectroscopy (CIS) with the objective to reveal the main structural motives of these complex species. The measured spectroscopic IR signatures of the monomer and dimer complexes serve to validate their structures, which are calculated and selected by quantum chemistry using an NN-based machine learning algorithm for conformational search.

## Method

Our experimental setup has been described elsewhere^25,31^ (see ESI,† for details). Briefly, the protonated monomer and oligomers of Phe_2_ are produced from solution using a nano-electrospray ion source (nano-ESI), mass-selected by a quadrupole mass filter and guided into a cold octupole ion trap, where they are collisionally cooled to *T*_vib_ ≃ 10 K.^32^ Once cooled, the ions are interrogated by UV or by IR and UV laser pulses to provide photofragmentation electronic and vibrational gain/depletion spectra, respectively.

To generate a sequence of unique low-energy conformers, we employed a modified machine learning method of the reference^33^ with a simplified neural network. The reinforced learning algorithm of proximal policy optimization (PPO) was used for training NN to generate a set of unique low-energy conformers (see ESI,† for details). To enhance the diversity of candidate structures, our NN was trained to predict all structures of the same size, regardless of the charge locations. The training continued until the advantage of the predicted trajectory reached a plateau. The NN then generated a large set of structures, whose uniqueness was assessed using the torsion fingerprint deviation method.^34^ The subsequent energy optimization of these structures with MD simulations yet yields an impractical for a detailed analysis number of unique conformers (*e.g.*, 10^4^ for Phe_2_ dimers). We, therefore, cluster the conformers to the families of similar structures, taking as the metric their patterns of hydrogen bonds and p–π interactions, which are known to play a crucial role in the self-assembly process, and the charge location. Retaining in each family only the lowest energy structures with the 10 kcal mol^−1^ cutoff provides a targeted pool of candidate conformers (*e.g.*, about 100 structures per each charge location of the Phe_2_ dimer). These conformers were, finally, optimized at the level of the density functional theory (DFT), and their vibrational spectra were calculated in the harmonic approximation (see ESI,† for details).

## Results and discussion

**Fig. 1:**
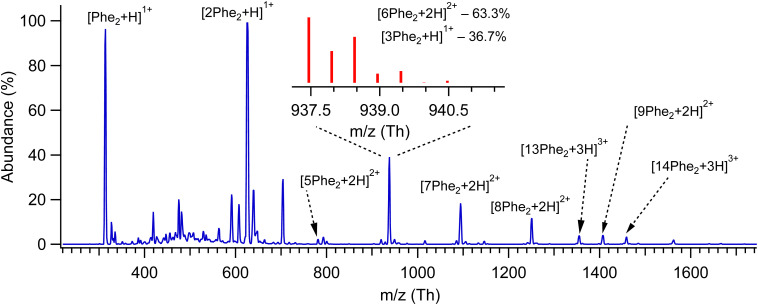
Mass spectrum of protonated non-covalent complexes of Phe_2_ measured with low-resolution quadrupole MS. The peaks are labelled by the respective prevailing complexes, as determined from ^13^C isotopic distribution, which have been measured additionally using HR MS; the insert shows such a distribution and the calculated relative concentrations of the assigned complexes for the peak around 938 Th.

Fig. 1 shows the mass spectrum of protonated Phe_2_ and its non-covalent complexes [*n*Phe_2_ + *z*H]^z+^ (*n* = 2–14, *z* = 1–3), generated by our nano-ESI source and recorded with a quadrupole mass spectrometer (Q1; see ESI†). Some of the complexes, although different in mass, have the same *m*/*z* value, which does not allow us to isolate them for individual spectroscopic sensing. Additional measurements with an Orbitrap-based high-resolution (HR) MS (Exactive, Thermo Fisher) provide the ^13^C isotopic structures of these peaks (Fig. S1, ESI†), which enables size assignment of the complexes. The two most abundant peaks at 313 Th and 625 Th correspond to singly protonated monomer and dimer, respectively, without any detectable contribution from higher charge states. Similarly, the peaks assigned to [7Phe_2_ + 2H]^2+^, [9Phe_2_ + 2H]^2+^ and [13Phe_2_ + 3H]^3+^ complexes exhibit no noticeable contaminations from other charge states, while the peak at 1250 Th is >90% due to the [8Phe_2_ + 2H]^2+^ octamer complex. The isotopic distribution of the peak at 938 Th (insert in Fig. 1) exhibits the presence of [3Phe_2_ + H]^1+^ and [6Phe_2_ + 2H]^2+^ complexes. The fit of the measured distribution by the sum of the distributions calculated for these ions suggests 33% and 67% contributions to this peak of the trimer and hexamer, respectively. The relative abundance of the singly protonated complexes in our experiment drops quickly from 100% for the dimer to 12%, 0.5% and below 10^−2^% for the trimer, tetramer and pentamer, respectively. In contrast, the abundance of the doubly protonated complexes increases rapidly from, at most, 2.8% for pentamer to 24% for the hexamer and then drops slowly (18.5%, 10.5% and 6.5% for the hepta-, octa- and nonamer, respectively; Fig. S2, ESI†). Although we do not speculate on the reasons for such a distribution, the fact that among the doubly protonated complexes, the hexamer appeared to be the most abundant may root from its particularly stable geometry.

**Fig. 2:**
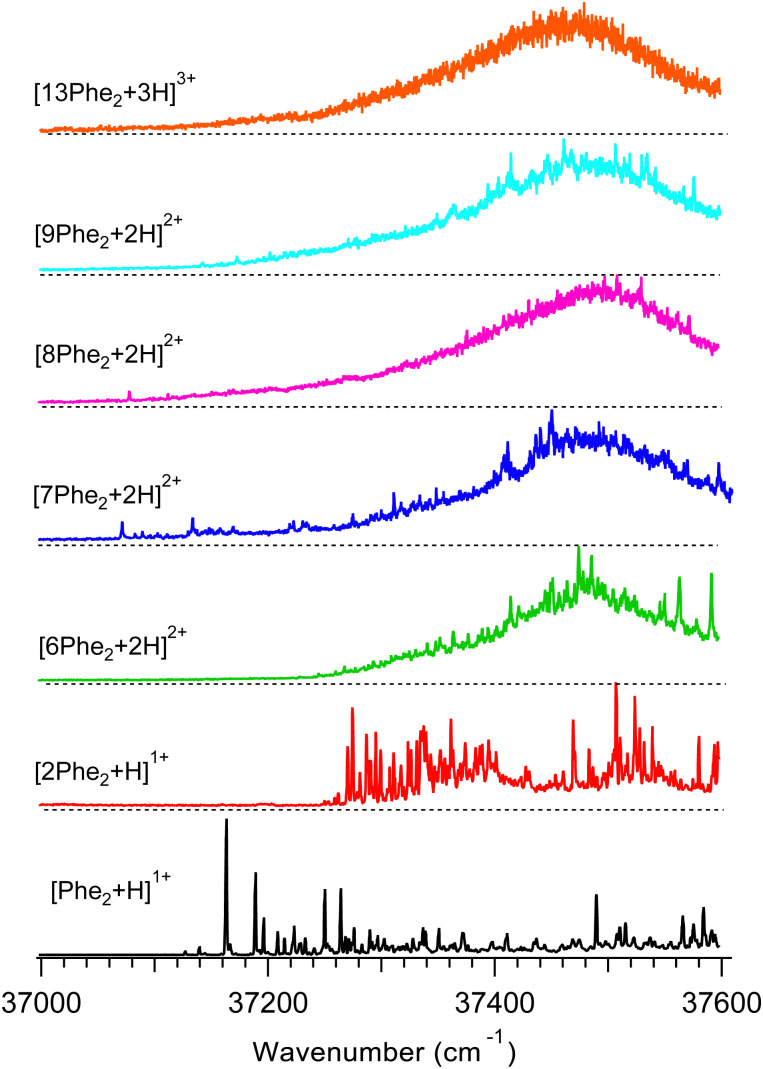
UV photofragmentation spectra of protonated Phe_2_ and its non-covalent complexes [*n*Phe_2_ + *z*H]^z+^ (*n* = 2, 6–9, 13; *z* = 1–3) measured in an octupole ion trap at *T* = 6 K. The spectra are labelled by the most abundant oligomers of the same *m*/*z* (Fig. 1).

Fig. 2 shows UV photo-fragmentation spectra of protonated Phe_2_ and its oligomers that appeared to be sufficiently abundant for the experimental interrogation. All spectra of the oligomers were measured by detecting each time the most abundant charged photofragments with *m*/*z* below that for the parent ions (Fig. S3, ESI†). For the produced 1 : 2 mixture of the trimer and hexamer isolated by the Q1 MS filter at *m*/*z* = 938 Th (see above), an additional UV spectrum was measured by detecting a specific to the hexamer only minor (1.7% yield) photofragment with *m/z* = 1250 Th (loss of 2Phe_2_^1+^). The near identity of this spectrum (Fig. S4, ESI†) to the respective trace in Fig. 2 implies that the prevailing (∼90%) contribution to the latter is, indeed, from the hexamer [6Phe_2_ + 2H]^2+^. This also suggests the 4.5 times higher photo-fragmentation yield into this channel for the hexamer than for the trimer.

The UV band origin in the spectrum of the monomer (37 163.3 cm^−1^) is unusually far redshifted from that of neutral (37 537 cm^−1^)^35^ and of protonated (37 529.6 cm^−1^)^25,36^ amino acid Phe. Although the substantial spectral shifts may result from a strong interaction of the protonated N-terminus with aromatic rings (p–π interaction), such large redshifts have not been observed in the peptides with single Phe residue. A similar, although smaller, redshift of the Phe band origin (37 261 cm^−1^) was measured in the gas phase for protonated pentapeptide [d-Ala^2^, d-Leu^5^]-enkephalin, whose validated structures exhibit two aromatic rings (of Phe and Tyr) stacked together around the protonated N-terminus.^37^ The shift could originate either from the excitonic interaction of the two aromatic rings or from the very strong/specific interaction of the Phe ring with the charge. To decouple the two possible cases for Phe_2_H^+^, we measured a UV spectrum (Fig. S5, ESI†) of singly protonated dipeptide Ala-Phe, whose structural flexibility should allow for its protonated N-terminus to interact with the single aromatic ring. The UV band origin of [Ala-Phe + H]^+^ appears, indeed, redshifted (37 138.4 cm^−1^) even more than that for Phe_2_H^+^. In contrast, for the reversed dipeptide, [Phe-Ala + H]^+^, in which the ring and the charge belong to the same (Phe) residue, the measured UV spectrum exhibits, eventually, no spectral shift of the band origin. This result is similar to the earlier observed, although smaller, redshifts for a pair of singly protonated dipeptides Ala-Tyr and Tyr-Ala.^27^ We may therefore conclude that the observed redshift in the spectra of Phe_2_H^+^ is, most likely, due to the interaction of the charge on the N-terminus with the chromophore of the C-terminal Phe. This structural constraint will be used later in validating the computed structures of Phe_2_H^+^.

The UV band origin of [2Phe_2_ + H]^+^ dimer is still substantially (∼230 cm^−1^) redshifted with respect to that of neutral Phe. This observation suggests that, although the p–π interaction in the dimer weakens, at least, one of the four Phe rings remains close to the protonated N-terminus of the complex. This conclusion can be used for rejecting the computed conformers that lack such structural features.

The UV spectra of the doubly charged species (from 6- to 9-mer) demonstrate similar shapes dominated by a broad band with a maximum at around 37 470–37 500 cm^−1^. Upon increasing the size of the oligomers, the broadening gradually washes out the resolved peaks, leaving none of them for the 13-mer. This smoothing of the main band, most likely, reflects the growing number of chromophores that reside in slightly different local environments and, potentially, the higher conformational heterogeneity of larger oligomers.

The position of the UV band maxima in the UV spectra of the 6- to 13-mers shown in Fig. 2 remains close to that of neutral and protonated Phe. This implies that most of the Phe aromatic rings do not interact with protonated amino groups. There is also a number of the substantially redshifted (down to ∼37 070 cm^−1^) low intense narrow peaks in the spectra of the 7- to 9-mers. The presence of such peaks indicates that a few chromophores interact with protonated amino groups in these oligomers even stronger than in the monomer. It is worth noting that, although the net charge of these oligomers is +2, the total number of the charges can be higher, if some of the Phe_2_ units of the complexes are zwitterions. We may propose that the high flexibility of the large oligomers, like the 7- to 9-mers, allows approaching some of their Phe rings to the charges (if not the same residue) for strong p–π interactions. In contrast, there are no peaks and any detectable absorption below ∼37 240 cm^−1^ for the hexamer. This observation implies the hexamer structures, where no single charge closely points to any chromophore unless they both belong to the same residue. This indirect constraint, perhaps, can assist in the future conformational search for the lowest energy structure of this oligomer.

**Fig. 3:**
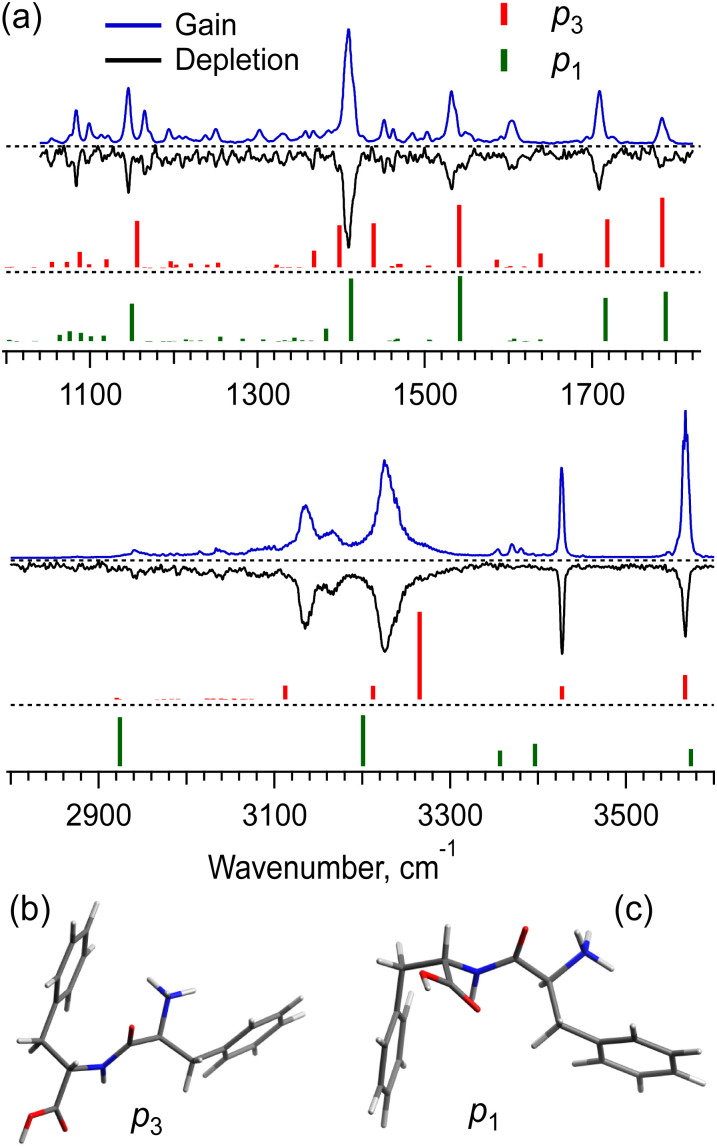
(a) IR gain and depletion spectra of [Phe_2_ + H]^+^ (blue and black traces) and computed vibrational transitions (color-coded vertical lines) for the conformer (b) p_3_ (0.4 kcal mol^−1^) and the most stable conformer (c) p_1_. The spectra were measured with a UV laser tuned to 37 156 and 37 163.3 cm^−1^. The calculated (in harmonic approximation) vibrational frequencies are scaled by the factors of 0.955 and 0.983 for 3 and 6 μm spectral regions, respectively.

The UV spectra of the monomer and dimer shown in Fig. 2 exhibit several well-resolved transitions, which enable conformer-selective IR spectroscopy of these complexes. The distinct partially resolved transition at 37 591cm^−1^ in the UV spectrum of the hexamer also allows for IR sensing of this large cluster. Fig. 3a shows the all-conformer “gain” and conformer-selective “depletion” IR spectra of [Phe_2_ + H]^+^, as well as the computed vibrational transitions for the two most relevant structures of the dipeptide. Overall, the two spectra look almost identical, which suggests the existence of only one major conformer of the protonated Phe_2_. A number of small peaks between 3340 and 3390 cm^−1^, tentatively, can be assigned to one or a few low abundant conformers. A comparison of the experimental IR spectra with the vibrational transitions calculated for the lowest-energy conformer p_1_ (Fig. 3c), reveals that this can be a minor, but not the major conformer. Indeed, it has a free NH of the amino group and an amide NH pointing towards one of the Phe rings, which reproduces the frequencies of the small peaks belonging to a minor conformer rather than the frequencies of the band at 3224.5 cm^−1^ and the narrow peak at 3427.1 cm^−1^. The conformational search identified, however, another conformer, p_3_ (Fig. 3b), which is only 0.4 kcal mol^−1^ higher in energy than the lowest one, but whose vibrational transitions match the experimental IR spectra much better. The p_3_ conformer has a very peculiar 3D structure, where the amino group is squeezed in a sandwich-like structure between two Phe rings^38^ and, in addition, forms a hydrogen bond with the carbonyl group, while the C-terminus and the amide NH are not involved in any intramolecular interactions. This accounts for a perfect match of the frequencies of free NH and OH vibrations and a reasonably reproduced triplet of the bound NH vibrations (the exact frequencies of the vibrations of H-bonded groups are known to be poorly calculated in harmonic approximation). In addition, and consistently with the above-derived UV spectral constraint, the p_3_ conformer exhibits a close proximity of its C-terminal aromatic ring to the protonated N-terminus, while the lowest energy conformer lacks this structural feature. Taken together, we consider the p_3_ conformer as the most abundant conformer present in our experiment. Consistently, a very similar structure was among the two candidate conformers, previously proposed in the cited above work of Lepere *et al.*,^27^ high spectral resolution in our experiments, finally, allows for unambiguous selection of the lowest energy structure. The successful assignment of the monomeric structure validates the NN-based conformational search procedure and provides a certain level of confidence in its use for finding structures of larger complexes.

**Fig. 4:**
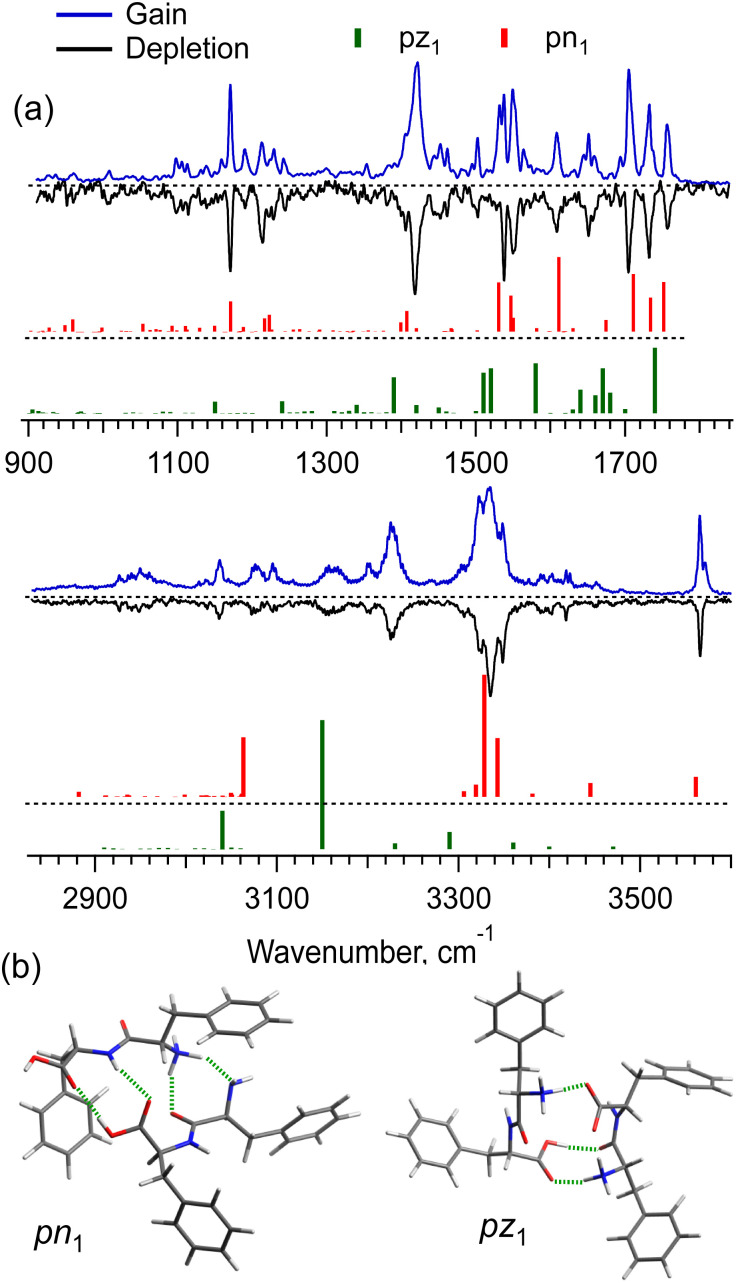
(a) IR gain and depletion spectra of singly protonated non-covalent complex [2Phe_2_ + H]^+^ (blue and black traces) measured with a UV laser tuned to the peaks at 37 241 and 37 274 cm^−1^, and the vibrational transitions (color-coded vertical sticks) calculated in harmonic approximation for the low potential energy structures (b) pn_1_ (0.4 kcal mol^−1^) and pz_1_ (0 kcal mol^−1^); intermolecular hydrogen bonds are shown in green.

The [*2*Phe_2_ + H]^+^ dimers may exist in the charge-solvated (a protonated–neutral pair, pn) and salt-bridge (a protonated–zwitterion pair, pz) forms. The presence of the two charge forms adds great complexity to both the conformational search and the conformational assignment of the measured IR spectra. Fig. 4a shows the IR gain and depletion spectra of the [2Phe_2_ + H]^1+^ dimers. A slight difference between the two spectra (*e.g.*, the sharper/better-resolved bands at *ca.* 3340 cm^−1^ and the disappearing transitions at 1531 and 3571 cm^−1^ in the depletion spectrum) suggests the presence of a highly abundant main conformer and a minor one with ≃2.5 times lower abundance. We cannot rule out the presence of other minor conformers that contribute to the measured spectra. An important observation is that the two most abundant (families of) conformers exhibit a transition assigned to free OH-stretch vibration. This fact allows us to select the pn structures as the most abundant ones under our experimental conditions. Indeed, Fig. 4a shows the vibrational transitions calculated for the most stable pz- and pn-type of structures (pz_1_ and pn_1_, respectively; Fig. 4b). While pn_1_ conformer owns an OH-group that is free of any non-covalent interactions, the zwitterion one does not. Moreover, with the energy cutoff of 10 kcal mol^−1^, none of the optimized pz structures exhibits this feature. Analysis of the two structures reveals that only in the pn_1_ conformer the charged N-terminus, although a bit remote (4.8 Å), points to the Phe ring of not the same residue. This is consistent with the observed (Fig. 2) substantial redshift of the band origin in the UV spectra of the dimer. We thus may suggest that the protonated Phe_2_ dimers in our experiment are present, mainly, as charge-solvated conformers.

Although the potential energy (including zero-point vibrational energy) of the pn_1_ conformer is higher than that of pz_1_, at an increased vibrational temperature, the former may become the most populated structure, if we account for the entropy of the dimers. Our detailed analysis (Fig. S6 and S7, ESI†) suggests that the strong intermolecular NH_3_^+^⋯COO^−^ electrostatic interaction in the salt-bridge complexes leads to their higher torsional rigidity. As a result, their entropy increases with temperature slower than that for the charge-solvated dimers. Above, approximately, 170 K, the free energy of pz_1_ becomes higher than that of pn_1_. This temperature would be consistent with the non-adiabatic nature of the fast collisional cooling of biomolecular ions in cryogenic traps. The conformational distribution of large ions promptly cooled from room temperature of a pre-trap to about 10 K should correspond to a temperature that is below but close to the former one.^39^ Overall, we cannot rule out the existence of other pn conformers of [2Phe_2_ + H]^+^, whose IR spectra match the experiment better than that of pn_1_, but which were lost in the conformational search among “myriads” of candidate structures. Nevertheless, the whole body of evidence allows us to conclude that the prevailing contribution to the observed spectra is by the charge-solvated but not by the zwitterion structures.

**Fig. 5:**
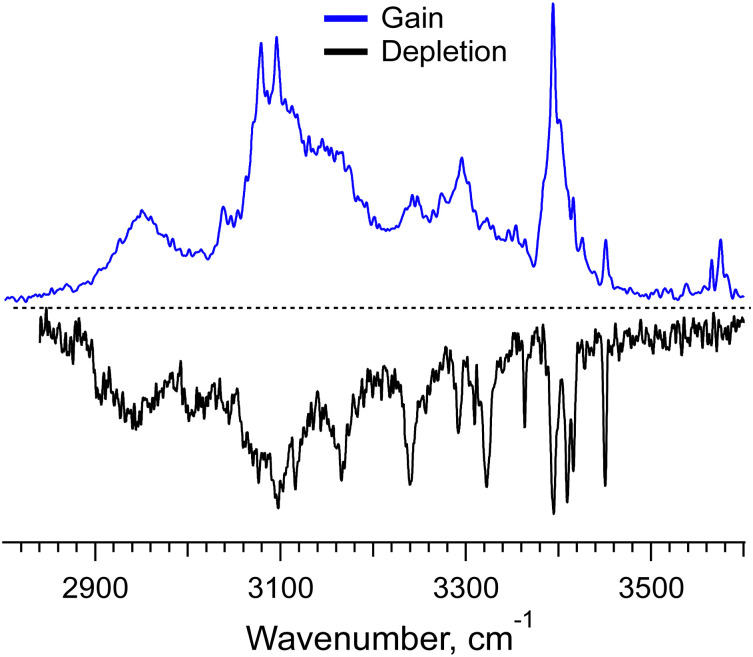
IR gain and depletion spectra of the doubly protonated non-covalent hexamer complex [6Phe_2_ + 2H]^2+^ measured with a UV laser tuned to 37 210 and 37 591.2 cm^−1^.

Even with the NN algorithms, the calculations at our affordable level of computational power and time become unfeasible for the doubly protonated hexamer of Phe_2_, for which we have measured a gain spectrum and even a conformer-selective depletion spectrum (Fig. 5). The depletion IR spectrum, which has been measured at the only nearly isolated prominent UV peak specific to [6Phe_2_ + 2H]^2+^, exhibits several well-resolved IR transitions. They correspond to the free NH- and/or weakly bound OH-stretches of this large system; there are no transitions of the free OH-stretches in this spectrum. These transitions appear, however, as weak peaks around 3585 cm^−1^ in the gain spectrum, which implies the presence of some other minor conformers. These few hints, perhaps, can be used in the forthcoming years upon further development of NN algorithms and affordable computational power to guide the accurate determination of intrinsic structures of such large flexible molecular systems.

## Conclusions

In conclusion, we interrogated protonated dipeptide [Phe_2_ + H]^+^ and its non-covalent complexes from dimer to 13-mer using UV and IR cold ion spectroscopy, HR mass spectrometry and quantum chemical calculations. UV spectroscopy suggests that, likely, there are no specific/strong p–π interactions in the complexes larger than 8-mers, which may imply some structural ordering of the oligomers. The computations, augmented by a neural network machine learning conformational search, solved the 3D structure of the [Phe_2_ + H]^+^ monomer and the [2Phe_2_ + H]^+^ non-covalent complex. The experimentally observed structures have been assigned through a comparison of their measured conformer-selective IR spectra with those computed for the candidate conformers. This conformational validation was further reinforced by the qualitative structural constraints derived from the UV spectra of the ions.

The structure of the monomer exhibits a stacking of the two aromatic rings around the charged N-terminus; the p–π coupling of the ring that belongs to the C-terminal Phe residue leads to the observed exceptionally large redshift of the UV band origin for this conformer.

Although the calculated most stable structure of the dimer is a zwitterion, the experimentally observed most abundant conformer was unambiguously assigned to the lowest energy charge-solvated structure. A characteristic of this conformer-free OH-stretch transition, observed in the IR spectrum, has not been found in any of the computationally optimized zwitterionic structures; the observed substantial redshift of the UV band origin also excludes the most stable zwitterion conformer, which exhibits p–π couplings only between the N-termini and the Phe rings of the same two residues. The high abundance of the pn-type conformers originates from their lower rigidity as compared with the zwitterionic structures. Already at the vibrational temperature of ions above ∼170 K the calculated entropy factor makes the free energy of the charged-solvated conformers lower than that of the zwitterions, which leads to lower abundance of the latter.

The lowest energy structures of the dimer have been found by the conformational search governed by the NN machine learning, which demonstrates its potential in structural determination for large molecular systems. The extension of the theory to the complexes larger than the dimer, with the available computational facilities did not look feasible, in particular, owing to the fact that the trimers and tetramers of Phe_2_ could not be experimentally isolated. High-resolution mass spectrometry has shown that the larger complex, for which the UV photo-fragmentation spectrum could be measured, is the doubly protonated hexamer. Pushing the limits of possible and yet ahead of the required structural calculations, we measured its conformer-selective IR spectrum. This unique data may serve in the future as a benchmark for validating the structure of the Phe_2_ hexamer, which, ironically, is a building unit for many self-assembled condensed phase supramolecular structures.

## Data availability

The data supporting this article have been included as part of the ESI.†

## Conflicts of interest

There are no conflicts of interest to declare.

## Supplementary Material

CP-026-D4CP03476G-s001
