# Supplementary material for: Oligomers of diphenylalanine examined using cold ion spectroscopy and neural network-based conformational search
Source: Phys Chem Chem Phys. 2024 Oct 16;26(44):27964–71. doi: 10.1039/d4cp03476g (PMC11525952; doi:10.1039/d4cp03476g)
Supplement: CP-026-D4CP03476G-s001 [file CP-026-D4CP03476G-s001.pdf]

Supplementary Information (SI) for Physical Chemistry Chemical Physics.
This journal is © the Owner Societies 2024

Supporting Information

Oligomers of Diphenylalanine Interrogated by Cold Ion Spectroscopy and Neural Network-based Conformational Search

Vladimit Kopysov, Ruslan Yamaletdinov, and Oleg V. Boyarkin*

SCI-SB-RB, ISIC, Ecole Polytechnique Fédérale de Lausanne, CH-1015 Lausanne, Switzerland

*Corresponding author: oleg.boiarkin@epfl.ch

Table of Contents

Experimental and Computational details S2

Figure S1. Low- and high-resolution MS of protonated oligomers of Phe_2 .... S5

Figure S2. Relative abundance of the Phe_2 protonated oligomers S6

Figure S3. Photofragment MS for protonated oligomers S7

Figure S4. Oligomer-selective UV fragmentation spectra of the trimer and hexamer S8

Figure S5. UV spectra of AlaPhe, Phe_2 and PheAla S9

Figure S6. Matrices of averaged distances in the protonated dimer of Phe_2 .... S10

Figure S7. Relationship between E_{ZPE} and $T{\cdot}S$ S11

Additional references S12

Experimental details

Protonated monomer and oligomers of Phe_2 are produced from solution using a nano electrospray ion source (nano-ESI), mass-selected by a quadrupole mass filter (Q1), and guided into a cold octupole ion trap maintained at T=6 K. The ions are trapped and cooled in the octupole by collisions with He buffer gas. Once cooled, the ions are interrogated by UV and IR laser pulses of 5-10 ns duration, delayed by 200 ns. The released ions are then detected with a second quadrupole mass spectrometer (Q2). In the single laser UV spectroscopy experiments Q2 was tuned in alternative cycles of measurements to m/z of a fragment or the parent ions to determine the relative UV photofragmentation yield. The cold trap was filled in and the UV laser fired at 20 Hz repetition rate, while the IR optical parametric oscillator fired at 10 Hz.

Double resonance IR-UV CIS uses vibrationally resolved transitions in UV photofragmentation spectra to label different conformers of cold ions. A pulse from an IR laser heats the corresponding conformer internally, causing an inhomogeneous broadening and a redshift of electronic transitions, which are monitored by a subsequent UV laser pulse. The IR-induced changes in UV absorption allow for measurements of two different types of spectra.

An IR "gain" spectrum is measured by fixing the UV laser wavenumber to the red from the band origin, where absorption by cold ions is negligible, and scanning IR laser wavenumber. IR pre-excitation of any conformer results in an increase in UV fragmentation. An IR gain spectrum thus reflects the IR transitions of all abundant conformers of the ion in a single IR wavenumber scan.

An IR "depletion" spectrum is measured by fixing the UV laser wavenumber at an electronic transition that is specific to one conformer of the studied ion. IR pre-excitation of the same conformer results in the drop (depletion) of the UV-induced photofragmentation. Measuring this depletion as a function of IR wavelength generates an IR spectrum of this conformer only.

L-Phe-*L*-Phe (≥98% purity, purchased from Bachem) was dissolved in water at a concentration of 1 mg/mL by sonicating the solution, heating it to 65 °C for 30 min, and gradually cooling it down to room temperature. Right before spraying, the solution was acidified with 0.2% of acetic acid.

Details of Computational method

During each step t of the generation process, the neural network (NN) takes the current structure (s_t) and its memory state as input. Utilizing this information, the NN proposes the next structure, which is then subjected to molecular dynamics (MD) simulations to refine the structure and obtain its energy.

After the MD optimization, each new structure is assigned a score based on its energy and uniqueness. If the proposed structure has already been observed in previous steps, its score is set to 0. Otherwise, $Score(s_{t+1}) = \exp[-(E(s_{t+1}) - E_0)/k_B\tau]/\,Z_0\,)$, where τ - model temperature, k_B is the Boltzmann constant, $E(s_{t+1})$ represents the energy of the proposed structure, and E_0 and Z_0 are the normalized score and energy,

respectively. After the conformer set reaches a maximum size of 20, the Generalized Advantage Estimation is used to evaluate its advantage.

In our work, the NN architecture consists of a multi-layer long short-term memory (LSTM) unit, which receives hidden states ($h_t, c_t \in \mathbb{R}^{\boldsymbol{M}}; \boldsymbol{M} = 128$), and a structural vector ($s_t \in \mathbb{R}^{\boldsymbol{N}}$) containing torsion angles, relative ion positions (in spherical coordinates), Euler's angles for relative orientation, and a charge code indicating the charge state.

The neural network components used in the architecture are as follows:

1. $a = \mathbf{MLP}^{\boldsymbol{N,M,M}}(s_t)$, where $\mathbf{MLP}^{\boldsymbol{N,M,M}}$ represents a multiple-layer perceptron with dimensions $\boldsymbol{N, M, M}$.

2. $b, h_{t+1}, c_{t+1} = \mathbf{LSTM}^{\boldsymbol{M}}(a, h_t, c_t)$

3. $V_t = \mathbf{MLP}^{\boldsymbol{2M,1}}(a,b)$: The critic network predicts the value function.

4. $A_t = \mathbf{MLP}^{\boldsymbol{2M,2M,2(N-1)}}(a,b)$: The actor network predicts the action probabilities.

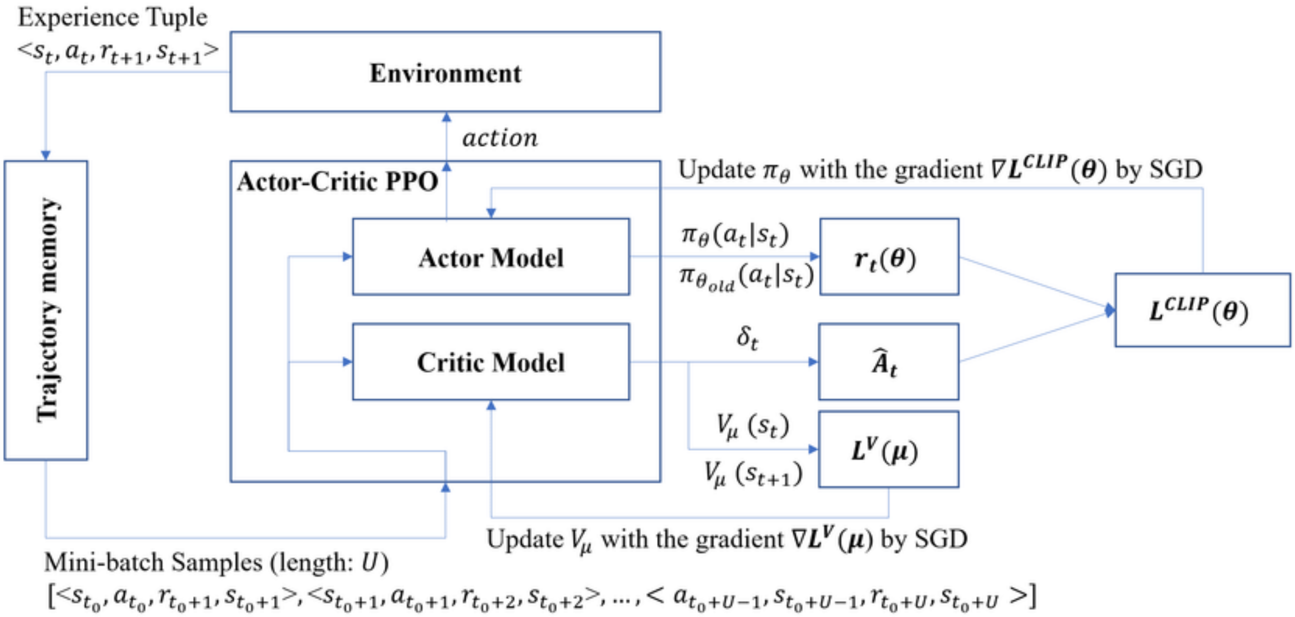

For clarity, the steps 1 and 2 were implemented and trained independently for both networks.

The training process of the neural network involves optimizing the parameters using Proximal Policy Optimization. We used a linear decrease of the temperature parameter τ from $\tau = 2000$ to 500K with a step size of 10K each epoch, and then held it constant until the advantage reaches a plateau. For all tasks involving the construction and training of neural networks, we employed the PyTorch package[1].

During training, we utilized batches with a size of 5 different conformers, each with a randomly selected charge state and initial structure s_0, but whith the same number of atoms.

The molecular dynamics (MD) simulations were performed using the OpenMM API for Python[2] [11], and Charmm 3.6 force fields for proteins[3] [12]. The number of optimization steps for minimizing the structures predicted by the neural network were limited to 100, while the subsequent optimizations were unconstrained. The machine learning generation of the initial pool of low-energy conformers was performed using a sequential conformer NN searcher. This algorithm is based on Proximal Policy Optimization (PPO) reinforcement learning in the rigid rotor approximation[4,5] [8,9]; the computations have been done using the in-house developed software suite. The DFT calculations were performed in Gaussian 16 package[6] [13] using B3LYP exchange-correlation functional[7] [14–16], 6-31G basis set with diffuse functions for heavy atoms and polarization functions for all atoms[8] [17,18].

Typically, NN and MD training took about 30 GPU hours both for monomers and dimers of Phe_2. DFT structural optimizations are more time consuming. It took, rough, 150 CPU per each of the 70 optimized structures. Finally, the frequency and free energy computations required almost 200 CPU and 500 CPU per each of the 30 selected monomer and dimer structures, respectively.

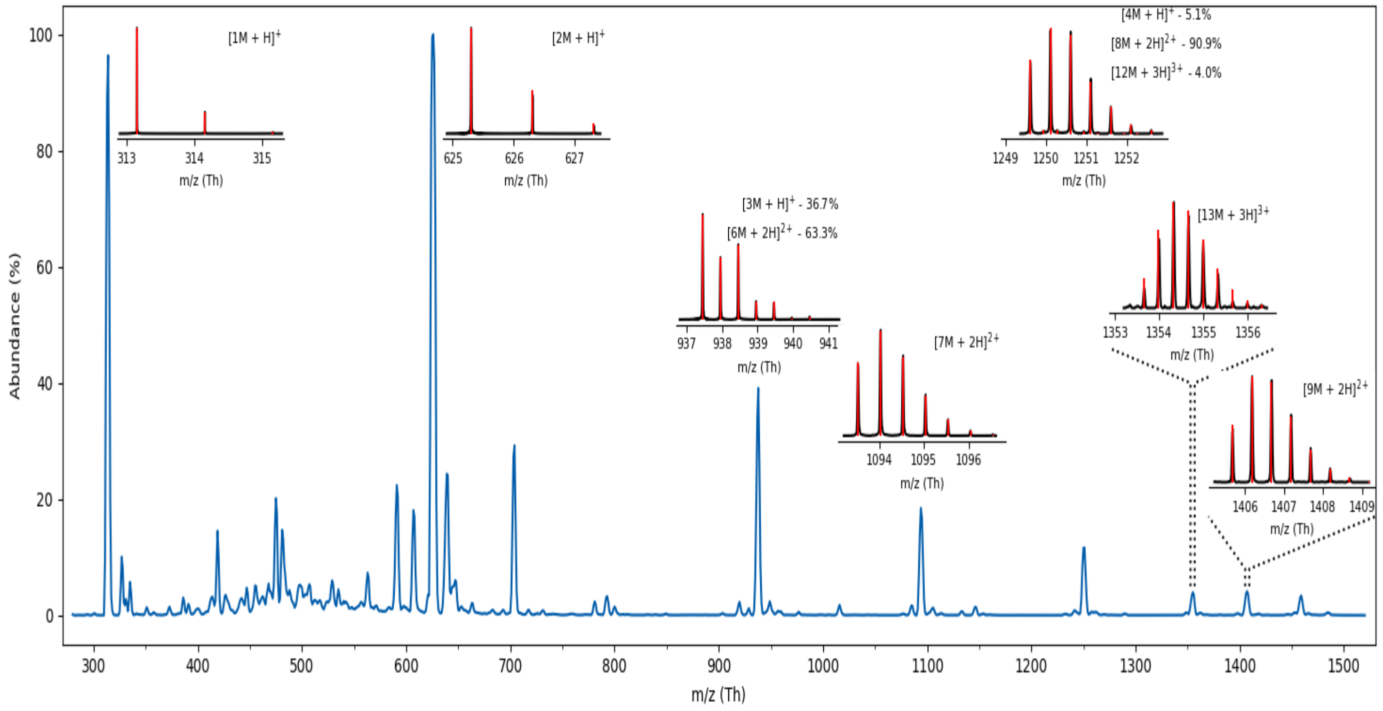

Figure S1. Mass spectrum of protonated Phe_2 mono- and oligomers, formed in electrospray ionization and measured by low-resolution quadrupole MS (blue trace). The inserts show the ^{13}C isotopic distribution of the respective peaks (black traces) measured with HR MS (Q-Exactive) and their fits (red vertical sticks) by combination of possible oligomers of the same m/z (see the labels above the inserts).

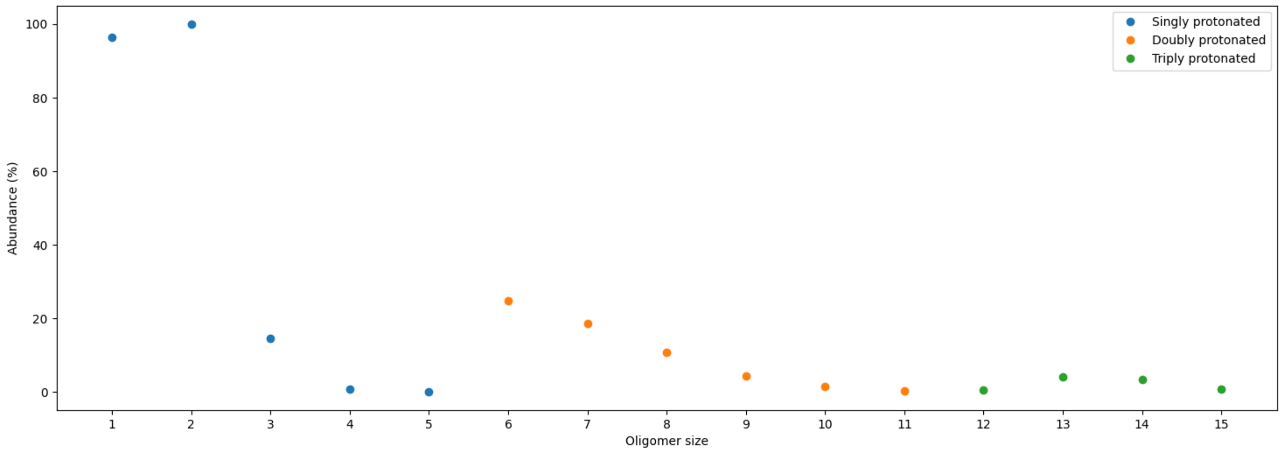

Figure S2. Abundance of the Phe_2 monomer and oligomers relative to the most abundant oligomer (n=2; 100%), as derived from the mass spectra in Fig. S1. Note the highest abundance of the hexamer among the oligomers larger than the dimer.

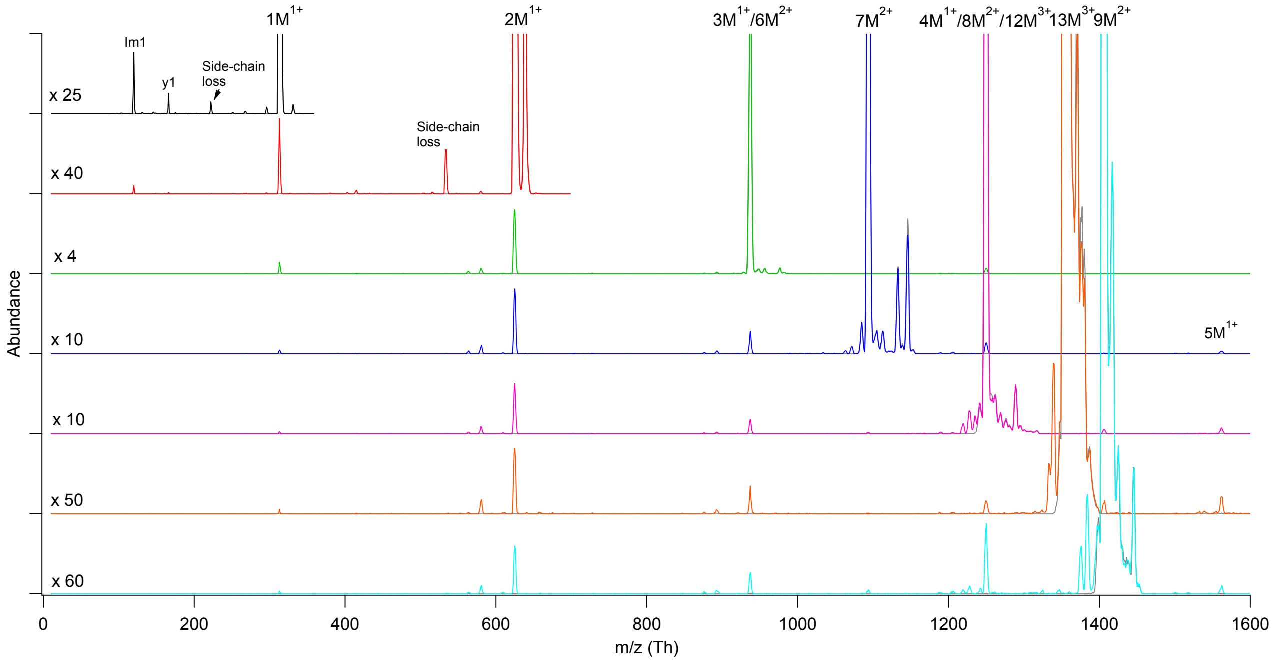

Figure S3. Photofragment MS for protonated oligomers of Phe_2.

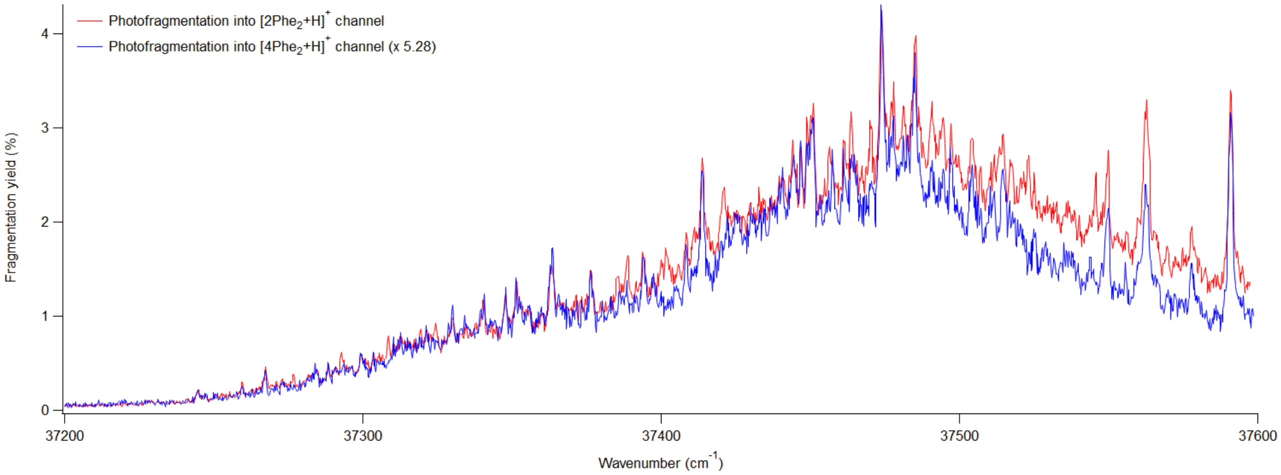

Figure S4. Oligomer-selective UV fragmentation spectra of the 1:2 mixture of $[3Phe_2+H]^+$ and $[6Phe_2+2H]^{+2}$ oligomers of the same m/z (see Fig S1), measured by detecting the $[2Phe_2+H]^+$ (red trace) and $[4Phe_2+H]^+$ (blue trace) fragments, specific for the trimer and hexamer, respectively.

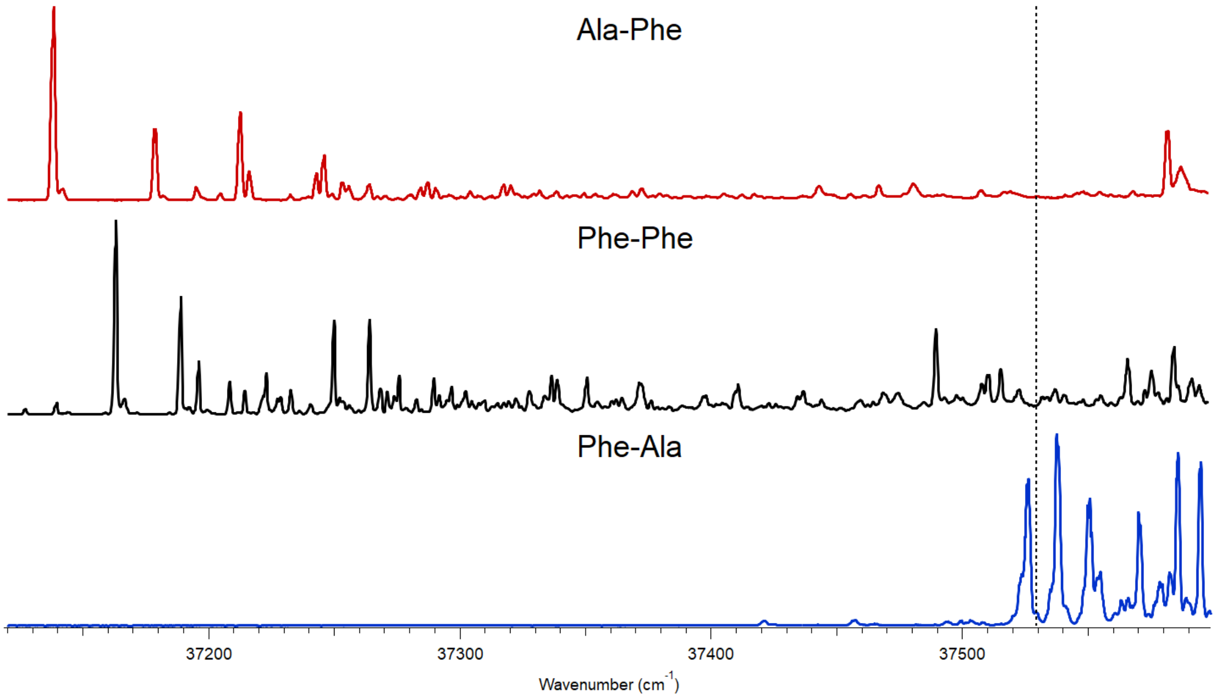

Figure S5. UV photo fragmentation spectra of singly protonated Ala-Phe (red), Phe_2 (black) and Phe-Ala dipeptides, demonstrating similarity in the redshifts of the band origins for the former two ions.

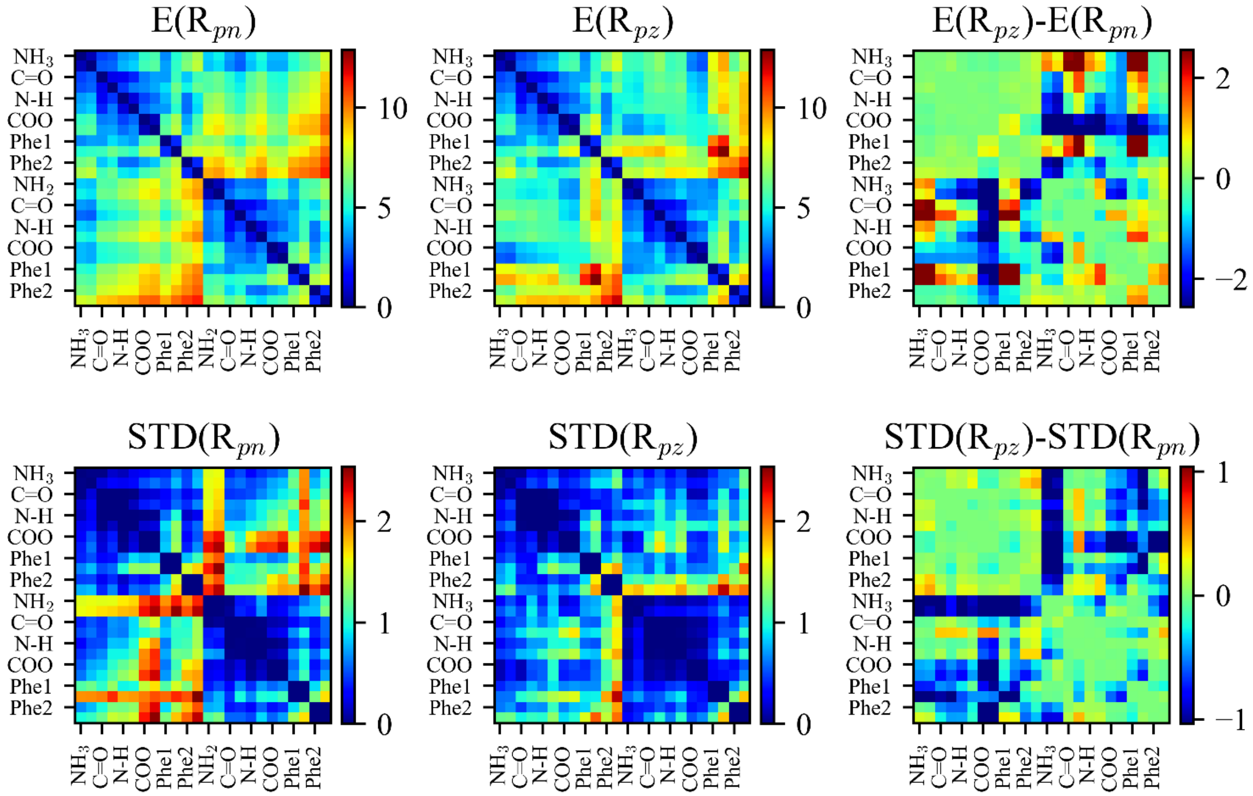

Figure S6. Averaged distance matrices (top) and their standard deviations (bottom) for $[2Phe_2+H]^+$ complex. From left to right: charge solvated form, zwitterionic and their differences. Specific groups are represented with two points: the first and second atoms (or the center position of the group if it consists of more than two atoms). One prominent observation is the pronounced structural diversity observed in the charge solvated form of Phe_2. In the zwitterionic form, strong Coulomb interactions, which predominantly govern the complex assembly, cause a more compact and predictable orientation of the charged groups.

Thermodynamical considerations

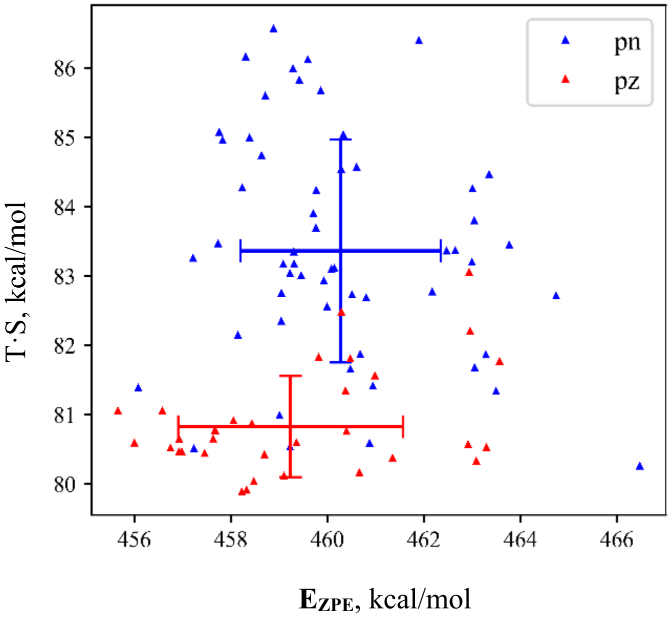

Fig. S7. The relationship between zero-point energy E_{ZPE} and $T \cdot S$ (at T=298) for calculated conformers families of the singly protonated Phe_2 dimers in the charge solvated (blue) and zwitterionic(red) forms. The the crosses indicate the mean values and the standard deviations of the corresponding data.

Additional references

1. A. Paszke et al., *PyTorch: An Imperative Style, High-Performance Deep Learning Library*, in *Advances in Neural Information Processing Systems 32* (Curran Associates, Inc., 2019), pp. 8024–8035.

2. P. Eastman et al., *OpenMM 7: Rapid Development of High Performance Algorithms for Molecular Dynamics*, PLOS Comput. Biol. **13**, e1005659 (2017).

3. J. Huang, S. Rauscher, G. Nawrocki, T. Ran, M. Feig, B. L. de Groot, H. Grubmüller, and A. D. MacKerell, *CHARMM36m: An Improved Force Field for Folded and Intrinsically Disordered Proteins*, Nat. Methods **14**, 71 (2017).

4. T. Gogineni, Z. Xu, E. Punzalan, R. Jiang, J. Kammeraad, A. Tewari, and P. M. Zimmerman, *TorsionNet: A Reinforcement Learning Approach to Sequential Conformer Search*, Adv. Neural Inf. Process. Syst. **33**, 20142 (2020).

5. R. Jiang, T. Gogineni, J. Kammeraad, Y. He, A. Tewari, and P. M. Zimmerman, *<scp>Conformer-RL</Scp> : A Deep Reinforcement Learning Library for Conformer Generation*, J. Comput. Chem. **43**, 1880 (2022).

6. Frisch, M. J.; Trucks, G. W.; Schlegel, H. B.; Scuseria, G. E.; Robb, M. A.; Cheeseman, J. R.; Scalmani, G.; Barone, V.; Petersson, G. A.; Nakatsuji, H.; Li, X.; Caricato, M.; Marenich, A. V; Bloino, J.; Janesko, B. G.; Gomperts, R.; Mennucci, B.; Hratchian, H. P.; Ortiz, J. V; Izmaylov, A. F.; Sonnenberg, J. L.; Williams-Young, D.; Ding, F.; Lipparini, F.; Egidi, F.; Goings, J.; Peng, B.; Petrone, A.; Henderson, T.; Ranasinghe, D.; Zakrzewski, V. G.; Gao, J.; Rega, N.; Zheng, G.; Liang, W.; Hada, M.; Ehara, M.; Toyota, K.; Fukuda, R.; Hasegawa, J.; Ishida, M.; Nakajima, T.; Honda, Y.; Kitao, O.; Nakai, H.; Vreven, T.; Throssell, K.; Montgomery Jr., J. A.; Peralta, J. E.; Ogliaro, F.; Bearpark, M. J.; Heyd, J. J.; Brothers, E. N.; Kudin, K. N.; Staroverov, V. N.; Keith, T. A.; Kobayashi, R.; Normand, J.; Raghavachari, K.; Rendell, A. P.; Burant, J. C.; Iyengar, S. S.; Tomasi, J.; Cossi, M.; Millam, J. M.; Klene, M.; Adamo, C.; Cammi, R.; Ochterski, J. W.; Martin, R. L.; Morokuma, K.; Farkas, O.; Foresman, J. B.; Fox, D. J. *Gaussian 16*, Revision C.01. Gaussian, Inc.: Wallingford, CT 2016.

7. A. D. Becke, *Density-Functional Exchange-Energy Approximation with Correct Asymptotic Behavior*, Phys. Rev. A **38**, 3098 (1988).

8. W. J. Hehre et al., *Self—Consistent Molecular Orbital Methods. XII. Further Extensions of Gaussian—Type Basis Sets for Use in Molecular Orbital Studies of Organic Molecules*, J. Chem. Phys. **56**, 2257 (1972).
